# Exposure of pregnant mice to triclosan impairs placental development and nutrient transport

**DOI:** 10.1038/srep44803

**Published:** 2017-03-21

**Authors:** Xinyuan Cao, Xu Hua, Xiaoli Wang, Ling Chen

**Affiliations:** 1 State Key Lab of Reproductive Medicine, Nanjing Medical University, Nanjing, 211166, China; 2 Department of Physiology, Nanjing Medical University, Nanjing, 211166, China; 3 Department of Pharmacology, Nanjing Medical University, Nanjing, 211166, China

## Abstract

Triclosan (TCS) is associated with spontaneous abortions and fetal growth restriction. Here, we showed that when pregnant mice were treated with 8 mg/kg TCS (8-TCS mice) on gestational days (GD) 6–18 fetal body weights were lower than controls. Placental weights and volumes were reduced in 8-TCS mice. The placental proliferative cells and expression of PCNA and Cyclin D3 on GD13 were remarkably decreased in 8-TCS mice. The decreases in activities and expression of placental System A amino acid or glucose transporters on GD14 and GD17 were observed in 8-TCS mice. Levels of serum thyroxine (T4) and triiodothyronine (T3) were lower in 8-TCS mice than those in controls. Declines of placental Akt, mTOR and P70S6K phosphorylation in 8-TCS mice were corrected by L-thyroxinein (T4). Treating 8-TCS mice with T4 rescued the placental cell proliferation and recovered the activity and expression of amino acid and glucose transporters, which were sensitive to mTOR inhibition by rapamycin. Furthermore, the replacement of T4 could rescue the decrease in fetal body weight, which was blocked by rapamycin. These findings indicate that TCS-induced hypothyroxinemia in gestation mice through reducing Akt-mTOR signaling may impair placental development and nutrient transfer leading to decreases in fetal body weight.

Triclosan (TCS), a broad-spectrum antimicrobial agent, is used in clinical settings and in various personal care and consumer products. TCS is one of the more frequently detected and highly concentrated contaminants in aquatic and terrestrial environments^1^. This compound has also been identified in the mother’s milk^2^,^3^. TCS was detected in 100% and 51% of urine and cord blood samples, respectively, obtained from 181 expectant mothers in New York^4^. High urinary TCS levels have been reported in spontaneous abortion patients^5^. Animal experiments have shown that the exposure of pregnant mice to TCS causes decreases in fetal body weight and viability^5^.

There are several biological activities of TCS that are unrelated to its antibacterial action. TCS has been demonstrated to be an inhibitor of estrogen sulfotransferase (EST)^6^. Exposure of pregnant mice to TCS (100 mg/kg) induces increases in activated E2 levels, leading to platelet aggregation and eventually placental thrombosis and necrosis^5^. Although the estrogen receptor (ER) antagonists can prevent the TCS-induced platelet aggregation and necrosis, the body weights of live fetuses are lower than those of control fetuses^5^. Thyroid hormones, which consist of triiodothyonine (T3) and thyroxine (T4), play a critical role in fetal growth and development. Dietary exposure to TCS in rats produces a dose-dependent decrease in serum T4 without any consistent change in TSH or T3^7^. Four-day oral administration of TCS in weanling female rats causes a dose-dependent decrease in circulating level of serum total T4^8^. The treatment with TCS decreases total serum T3 and T4 in pregnant rats and mice^5^,^9^. Published data consistently document a relationship between maternal thyroid hormones and feto-placental development. Maternal thyroid deficiency is associated with intrauterine growth restriction, fetal distress and low birth weight^10^,^11^. Treatment of pregnant rats with TCS (10 mg/kg) decreases the levels of total serum T4 and T3 and the pup body weights^9^. However, the underlying mechanisms have not yet been fully elucidated.

Several lines of evidence suggest that the thyroid hormones are critical for placental development. In mammals, the major determinant of intrauterine growth is the delivery of nutrients to the fetus *via* the placenta, which occurs primarily by diffusion and transporter-mediated transport. Placenta nutrient transport is dependent on placental size, morphology (exchange zone surface area and tissue thickness), nutrient transporter capacity/availability, and utero- and feto-placental blood flow^12^,^13^. Reduced placental development is associated with impaired intrauterine growth in experimental animals^14^. The capacity of the placenta to deliver nutrients from the mother to the fetus is dependent on the expression and function of nutrient transporters located in the placental barrier^12^. The Na^+^-dependent system A amino acid transporter (SNAT) transports small, neutral and nonessential amino acids across the placenta, while specific leucine transporter (system L) and taurine transporter (TAUT) usually transport the essential amino acids across the placenta^15^. As amino acids are the primary stimulus for insulin secretion by the fetal pancreas, there may be a direct link between placental amino acid transporter activity and fetal growth. The glucose transporter GLUT1 is the primary isoform involved in the transplacental movement of glucose^16^. Thyroid hormone may modulate the expression of GLUT1 and the translocation of GLUT1 protein into membranes^17^. The activation of thyroid hormone receptors is known to trigger the phosphatidylinositol 3-kinase (PI3K)/Akt-mTOR and ERK signaling pathways^18^,^19^. The Akt-mTOR and ERK signaling pathways have been shown to be involved in the placental growth, and placental amino acid transporter function and expression, as well as facilitative GLUT1 membrane localization^20^,^21^,^22^. Therefore, investigating whether TCS-induced reduction in thyroid hormones during pregnancy affects placental development and function is of great interest to us.

The administration of TCS on gestational days (GD) 1–3 has been observed to impair the blastocyst implantation in mice^23^. To investigate the influece of TCS on placental development and fetal growth, the pregnant mice were treated daily with 1, 4 or 8 mg/kg TCS from GD6 to GD18. We examined fetal viability and body weight, placental morphological structure and activities and expression of placental System A amino acid or glucose transporters during the exposure to TCS. To explore the underlying mechanisms, we furhter investigated the involvement of TCS-induced hypothyroxinemia in Akt-mTOR-P70S6K and ERK signaling pathways and placental development and function. Our results indicate that TCS-induced hypothyroxinemia impairs placental development and nutrient transfer through down-regulation of Akt-mTOR signaling pathway, leading to decreases in fetal body weight.

## Results

### Exposure of pregnant mice to TCS causes decreases in fetal body weight

**Figure 1:**
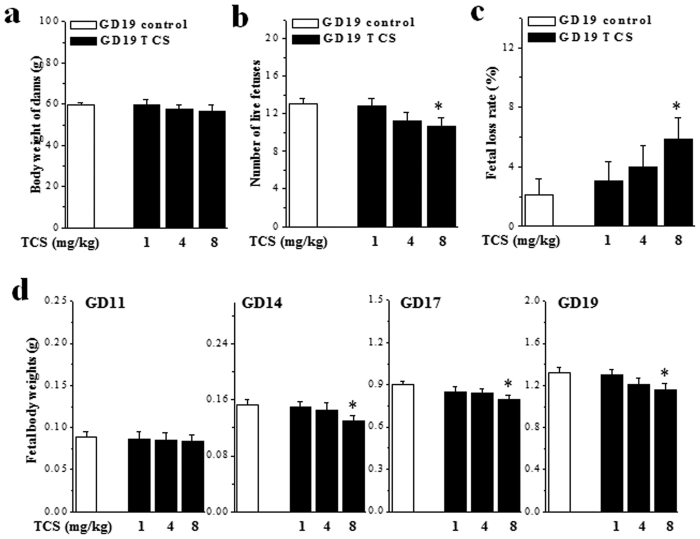
Exposure of pregnant mice to TCS causes decreases in fetal body weight. (**a**) Body weights (g) of pregnant mice on GD19. (**b**) Numbers of live fetuses in GD19 control mice and TCS mice. *P < 0.05 *vs*. control mice (one-way ANOVA). (**c**) Ratio of fetal loss to the total number of fetuses on GD19. *P < 0.05 *vs*. control mice (one-way ANOVA). (**d**) Body weights (g) of live fetuses on GD11, GD14, GD17 and GD19. *P < 0.05 *vs*. control mice (one-way ANOVA).

Pregnant mice were treated with the oral administration of 1, 4 and 8 mg/kg/day TCS (termed 1-TCS, 4-TCS and 8-TCS mice) from GD6 to GD18. The body weights of TCS mice on GD19 were not different from those of control mice, irrespective of dosage (*P* > 0.05, n = 10; Fig. 1a). In comparison with control mice (mean 13.1 ± 0.58/dams) on GD19, the numbers of live fetuses were slightly decreased in 8-TCS mice (10.7 ± 0.93, *P* < 0.05, n = 10 dams; Fig. 1b), but not in 1-TCS mice (12.8 ± 0.81, *P* > 0.05) or 4-TCS mice (11.3 ± 0.91, *P* > 0.05). The ratio of fetal loss in 8-TCS mice was higher than that in control mice (*P* < 0.05, n = 10; Fig. 1c). In addition, the declines in the body weights of the live fetuses (n = 10 dams) beginning on GD14 were observed in 8-TCS mice (GD11: *P* > 0.05; GD14: *P* < 0.05; GD17: *P* < 0.05; GD19: *P* < 0.05; Fig. 1d), but not in 1-TCS mice and 4-TCS mice (GD11-GD19: *P* > 0.05).

### TCS reduces placental size and cell proliferative activity

**Figure 2:**
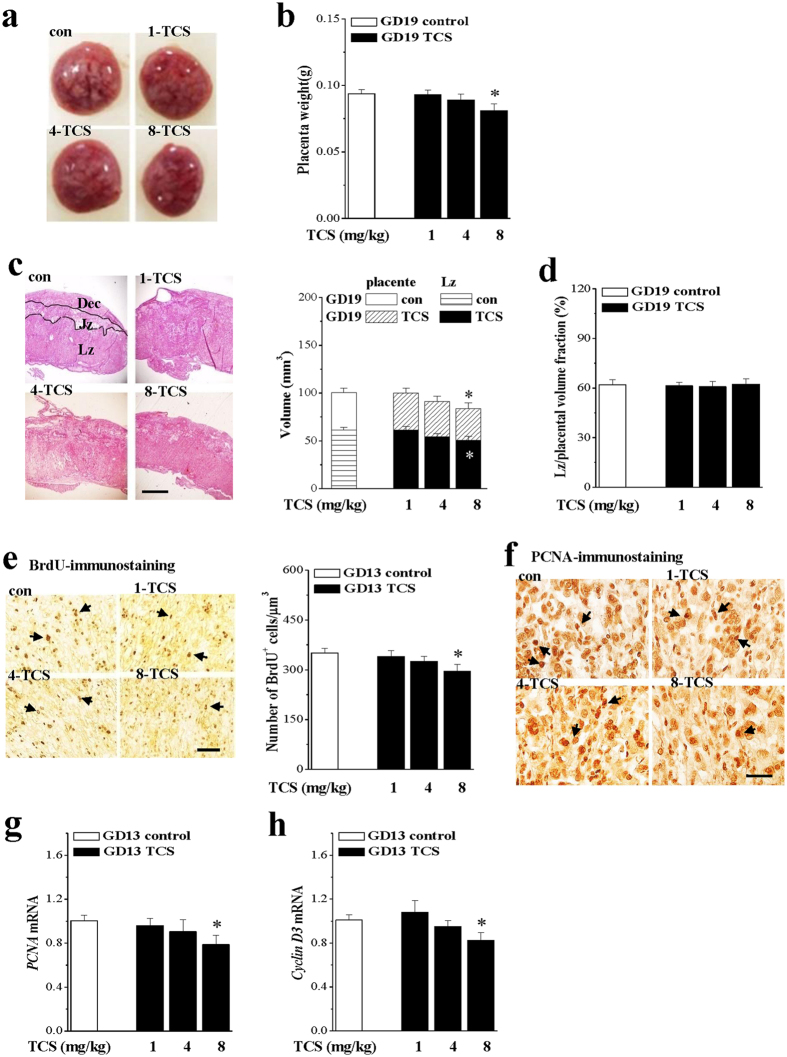
TCS reduces placental cell proliferation. (**a**) Representative picture of placentas in GD19 control mice and TCS mice. (**b**) Placental weight (g) on GD19. *P < 0.05 *vs*. control mice (one-way ANOVA). (**c**) Representative picture of placentas stained with hematoxylin and eosin on GD19. Lz: labyrinthine zone; Jz: junctional zone; Dec: deciduas. Scale bars = 200 μm. Bar graph indicates the placental volume and labyrinth zone volume on GD19. *P < 0.05 *vs*. control mice (one-way ANOVA). (**d**) Bar graph represents the volume fraction of the labyrinth zone relative to the volume of the entire placenta on GD19. (**e**) Representative picture of BrdU immunostaining on GD13. Bar graph represents the number of BrdU^+^ cells (black arrows) in the labyrinth zone. Scale bars = 50 μm. *P < 0.05 *vs*. control mice (one-way ANOVA). (**f**) Representative picture of PCNA immunostaining on GD13 in the labyrinth zone. PCNA^+^ cells are indicated by black arrows. Scale bars = 20 μm. (**g**,**h**) Bars indicate the level of placental *PCNA* and *Cyclin D3* mRNA on GD13. *P < 0.05 *vs*. control mice (one-way ANOVA).

The placenta is essential for normal embryonic development in the uterus, thus we examined placental weights and morphologies in TCS mice. The placental sizes (Fig. 2a) and the placental weights (*P* < 0.05, n = 20 placentas/10 dams; Fig. 2b) of GD19 8-TCS mice were less than those of control mice. Additionally, the stereohistological analysis revealed that overall placental volumes (*P* < 0.05, n = 20/10; Fig. 2c) and labyrinth zone (Lz) volumes (*P* < 0.05, n = 20/10) were significantly reduced in 8-TCS mice compared to control mice. There was no difference in the ratio of the volume of the labyrinth zone relative to the volume of the entire placenta between control and 8-TCS mice (*P* > 0.05, n = 20/10; Fig. 2d). We did not observe the placental thrombi or hemorrhaging, or tissue necrosis in TCS mice. By contrast, the placental weight (*P* > 0.05, n = 20/10), placental volumes (*P* > 0.05, n = 20/10) and labyrinth zone volumes (*P* > 0.05, n = 20/10) of 1-TCS mice and 4-TCS mice were not different from those of control mice.

During rat and mouse placental development, cell proliferative activity peaks on GD13 and then decreases during late gestation^24^. Proliferating cell nuclear antigen (PCNA) expression in rat placentas is very strong on GD13-17 followed by a gradual decrease on GD19-21^25^. To evaluate the influence of TCS on placental cell proliferative capability, we counted the BrdU-positive (BrdU^+^) cells and examined PCNA immunostaining and expression of PCNA and Cyclin D3 on GD13. We observed that the numbers of BrdU^+^ cells in the labyrinth zone of 8-TCS mice were decreased by approximately 16% compared to control mice (*P* < 0.05, n = 10 placenta/10 dams), while the numbers of BrdU^+^ cells in the labyrinth zone of 1-TCS mice and 4-TCS mice were unchanged (*P* > 0.05, n = 10/10). As shown in Fig. 2f, either PCNA immunostaining intensity or PCNA-positive (PCNA^+^) cells in the labyrinth zone of 8-TCS mice was obviously reduced in comparison with control mice (n = 10/10). Furthermore, the levels of placental *PCNA (P* < 0.05, n = 10/10; Fig. 2g) and *Cyclin D3* mRNA (*P* < 0.05, n = 10/10; Fig. 2h) in 8-TCS mice were lower than those in control mice, but in 1-TCS mice and 4-TCS mice did not differ from control mice (*P* > 0.05, n = 10/10).

### TCS reduces placental amino acid and glucose transporters activity and expression

The materno-fetal transfer of [^14^C]-methylaminoisobutyric acid (MeAIB), a non-metabolizable amino acid analogue that is usually transported across the placenta *via* SNAT, was increased on GD16^26^. Glucose is transported across the placenta by glucose transporters GLUT1 and GLUT3 on GD16^27^. To test whether the exposure to TCS affects the function of placental transporter system leading to the decrease in the body weights of the fetuses, we measured the function of placental SNAT and glucose transporters on GD14 and GD17, respectively, using unidirectional maternal-fetal [^14^C]-MeAIB and [^14^C]-methyl-D-glucose transfer. As shown in Table 1, the activity of SNAT per gram of placenta was significantly reduced in 8-TCS mice compared to control mice (GD14: *P* < 0.05, n = 50 fetuses/5 dams; GD17: *P* < 0.01, n = 50/5), but in 4-TCS mice and 1-TCS mice was significantly altered (*P* > 0.05, n = 50/5). In addition, the activity of glucose transporter per gram of placenta was lower in 8-TCS mice than in control mice (GD14: *P* < 0.05, n = 50/5; GD17: *P* < 0.05, n = 50/5). At all time points, [^14^C]-MeAIB (*P* > 0.05, n = 50/5) and [^14^C]-methyl-D-glucose accumulation per gram of fetus (*P* > 0.05, n = 50/5) in 8-TCS mice was the same as the control mice, indicating that the fetuses were receiving the appropriate amounts of radioactive label for their size.

We then analyzed the expression of the three isoforms of the SNAT family (Slc38a1/SNAT1, Slc38a2/SNAT2 and Slc38a4/SNAT4) and the two isoforms of the glucose transporter family (Slc2a1/GLUT1 and Slc2a3/GLUT3) on GD14 and GD17 (Table 2). The results showed that the levels of *SNAT1* (GD14: *P* < 0.05, n = 20/10; GD17: *P* < 0.01, n = 20/10) and *SNAT4* mRNA (GD14: *P* < 0.05, n = 20/10; GD17: *P* < 0.01, n = 20/10) were reduced in 8-TCS mice compared to control mice, while the level of *SNAT2* was not different between control mice and 8-TCS mice (*P* > 0.05, n = 20/10). In addition, the level of *GLUT1* mRNA (GD14: *P* < 0.05, n = 20/10; GD17: *P* < 0.05, n = 20/10) but not *GLUT3* mRNA (*P* > 0.05, n = 20/10) was lower in 8-TCS mice than that in control mice.

### TCS reduces thyroid hormones and reproductive hormones levels

Exposure to TCS has been reported to decrease serum total T4 and T3 levels in pregnant rats and mice, which affects fetal growth and development^5^,^9^. We measured the levels of pituitary-thyroid and gonadal hormones on GD17 (Table 3). In comparison with controls, 8-TCS mice exhibited reduced total T3 (*P* < 0.01, n = 10) and T4 levels (*P* < 0.01, n = 10) and slightly elevated TSH levels (*P* < 0.05, n = 10). There were no significant differences in the levels of serum E2 between control mice and TCS mice (*P* > 0.05, n = 10). Although 8-TCS mice tended to exhibit reduced P4 levels compared to control mice, the difference between the two groups failed to reach statistical significance (*P* > 0.05, n = 10). By contrast, the levels of thyroid hormones and reproductive hormones in 1-TCS mice (*P* > 0.05, n = 10) and 4-TCS-mice (*P* > 0.05, n = 10) were not different from those in control mice.

### TCS-induced hypothyroxinemia attenuates placental Akt-mTOR-p70S6K signaling

**Figure 3:**
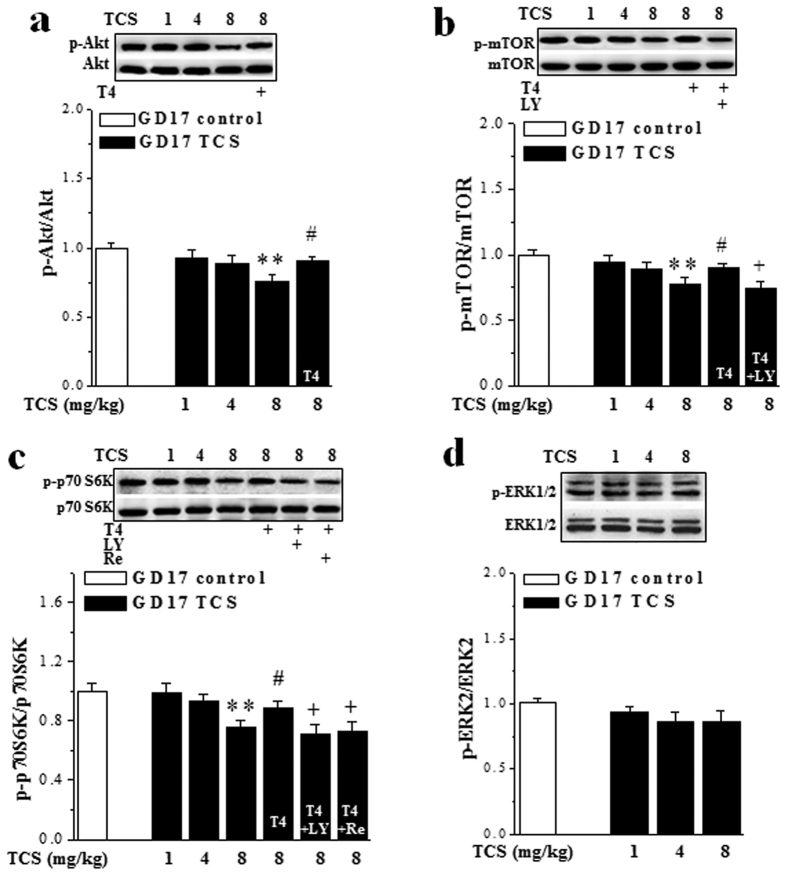
TCS reduces placental Akt-mTOR-p70S6K signaling pathway on GD17. (**a**–**d**) Bar graphs show the levels of phosphor-Akt, phosphor-mTOR, phosphor-p70S6K and phosphor-ERK2 in control mice, TCS mice and 8-TCS mice treated with T4 or T4+LY294002 (LY) or T4+repamine (Re). **P < 0.01 *vs*. control mice; ^#^*P* < 0.05 *vs*. 8-TCS mice; ^+^*P* < 0.05 *vs*. 8-TCS mice treated with T4 (two-way ANOVA). The blots in panel band were performed on the same blot membranes and shown as cropped images. Full-length versions of all western blots are presented in Supplementary Fig. 3.

Thyroid hormones stimulate the Akt-mTOR-p70S6K and ERK signaling pathways, which can regulate the activation of placental amino acid transporters^22^. Therefore, we examined the levels of placental Akt (phosphor-Akt), mTOR (phosphor-mTOR), p70S6K (phosphor-p70S6K) and ERK1/2 (phosphor-ERK1/2) phosphorylation on GD17. We observed that the levels of phosphor-Akt (*P* < 0.01, n = 20 placenta/10 dams; Fig. 3a), phosphor-mTOR (*P* < 0.01, n = 20/10; Fig. 3b) and phosphor-P70S6K (*P* < 0.01, n = 20/10; Fig. 3c) were reduced in 8-TCS mice, but not in 1-TCS mice (*P* > 0.05, n = 20/10) or 4-TCS-mice (*P* > 0.05, n = 20/10). Importantly, treating 8-TCS mice with L-thyroxinein (T4) from GD15 to GD17 corrected the above reductions in the levels of phosphor-Akt (*P* < 0.05, n = 20/10), phosphor-mTOR (*P* < 0.05, n = 20/10) and phosphor-p70S6K (*P* < 0.05, n = 20/10). These effects were blocked by the intrauterine injections of the PI3K inhibitor LY294002 on GD17 (*P* < 0.05, n = 20/10). Moreover, the T4-recovered phosphor-P70S6K was sensitive to the injection of rapamycin on GD15-17 (*P* < 0.05, n = 20/10). By contrast, no changes in the levels of phosphor-ERK1/2 were observed in TCS mice compared to control mice (*P* > 0.05, n = 20/10; Fig. 3d).

### Involvement of TCS-suppressed mTOR signaling in placental development and function

**Figure 4:**
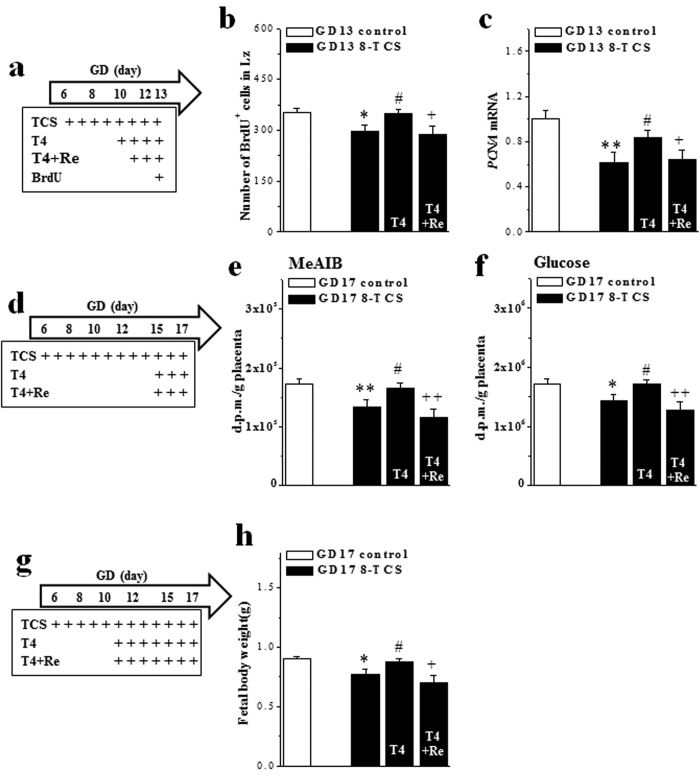
Involvement of TCS-induced hypothyroxinemia in placental development and function. (**a**,**d**,**g**) Time chart of the experimental procedures. + : time of drug administration. (**b**,**c**) Bar graphs represent the number of BrdU^+^ cells in the labyrinth zone and the level of placental *PCNA* mRNA on GD13 in control mice, 8-TCS mice and 8-TCS mice treated with T4 or T4+repamine (Re). *P < 0.05 and **P < 0.01 *vs*. control mice; ^#^*P* < 0.05 *vs*. 8-TCS mice; ^+^*P* < 0.05 *vs*. 8-TCS mice treated with T4 (two-way ANOVA). (**e**,**f**) Bar graphs represent the mean transfer of [^14^C]MeAIB and [^14^C]methyl-D-glucose across the placenta within a litter in d.p.m. (g placenta). *P < 0.05 and **P < 0.01 *vs*. control mice; ^#^*P* < 0.05 *vs*. 8-TCS mice; ^++^*P* < 0.01 *vs*. 8-TCS mice treated with T4 (two-way ANOVA). (**h**) Bar graph represents the body weights (g) of live fetuses on GD17. *P < 0.05 *vs*. control mice; ^#^*P* < 0.05 *vs*. 8-TCS mice; ^+^*P* < 0.05 *vs*. 8-TCS mice treated with T4 (two-way ANOVA).

Similar to the above findings regarding T4-recovered Akt-mTOR-p70S6K signaling, treating 8-TCS mice with the T4 replacement from GD10 to GD13 (Fig. 4a) corrected the reductions in the numbers of BrdU^+^ cells (*P* < 0.05, n = 20 placenta/10 dams; Fig. 4b) and the levels of *PCNA* mRNA (*P* < 0.05, n = 20/10; Fig. 4c). These effects were blocked by rapamycin (*P* < 0.05, n = 20/10). Interestingly, the T4 replacement on GD15-17 (Fig. 4d) rescued the activity of amino acid transporter (*P* < 0.05, n = 20/10; Fig. 4e) and glucose transporter (*P* < 0.05, n = 20/10; Fig. 4f) in 8-TCS mice. These effects of T4 were dependent on the activation of mTOR ([^14^C]-MeAIB: *P* < 0.01, n = 20/10; [^14^C]-methyl-D-glucose: *P* < 0.05, n = 20/10). Consistent with these findings, the treatment with T4 recovered the expression of SNAT1 (*P* < 0.05, n = 20/10; Table 2) and SNAT4 (*P* < 0.05, n = 20/10) in 8-TCS mice. And, these effects of T4 were blocked by rapamycin (*P* < 0.05, n = 20/10). The levels of *GLUT1* mRNA in 8-TCS mice were increased by the administration of T4 (*P* < 0.05, n = 20/10), but this change was not affected by rapamycin (*P* > 0.05, n = 20/10). Furthermore, the treatment of 8-TCS with T4 from GD10-17 (Fig. 4g) could rescue the decrease in fetal body weight (*P* < 0.05, n = 10 dams; Fig. 4h), which was sensitive to rapamycin (*P* < 0.05, n = 10).

## Discussion

The present study has provided the morphological and functional evidence that exposure to 8 mg/kg TCS in pregnant mice affects placental development and placental nutrient transport, leading to decreases in fetal body weight.

By examined seven tissues (placenta, liver, kidney, ovary, adrenal, spleen, and fat) of pregnant rats exposed to 30–600 mg/kg/day TCS, Feng *et al*. found the greatest bioaccumulation of TCS in the placenta^28^. Wang *et al*. recently reported that the levels of urinary TCS in partial spontaneous abortion patients (11.21 ng/ml) are higher than in normal pregnancies (0.99 ng/ml)^5^. Mice treated with 10 mg/kg/day TCS exhibit urinary TCS levels equivalent to those of abortion patients with high-exposure to TCS. The above dose of TCS has been reported to produce hypothyroxinemia in pregnant rats and mice^5^,^9^. Our results in the present study indicate that treating pregnant mice with TCS at a dose of 8 mg/kg elicits approximately 24% and 17% decreases in serum total T4 and T3 levels. Low thyroxine levels exert a positive feedback effect on thyroid-releasing hormone (TRH) leading to an increase in the TSH secretion. Decreases in serum T4 levels were observed in rats exposed to 35 mg/kg body weight TCS^29^ and in mice treated with 27 mg/kg body weight TCS^30^. We observed that the exposure of non-pregnant female mice to TCS (10 and 100 mg/kg/day) for consecutive 14 days caused the decline of serum T3 and T4 levels with the derangement of estrous cycle (unpublished data). The exposure to TCS for a short time (1 h) concentration-dependently decreases the sodium/iodide symporter (NIS)-mediated iodide uptake in a non-competitive manner^31^. TCS inhibits the activity of thyroid peroxidase, a critical protein involved in thyroid hormones synthesis^31^. In addition, TCS-induced hypothyroxinemia in rats may be partially caused by hepatic catabolism up-regulation facilitated by increases in pentoxyresorufin-O-deethylase (PROD) activity^32^. On the other hand, the exposure to 300–600 mg/kg TCS from GD6 to GD20 has been reported to reduce the levels of serum reproductive hormones P4, E2, human chorionic gonadotropin (hCG) and prolactin (PRL)^28^. Decreases in reproductive hormones P4 and β-HCG elicited by 100 mg/kg TCS in pregnant mice are thought to be due TCS-induced placental thrombi and hemorrhaging, or tissue necrosis^5^. By contrast, exposing pregnant mice to 8 mg/kg TCS failed to cause the placental thrombi or hemorrhaging (Supplemental Fig. 1) and the changes in the serum P4 and E2 levels. The exposure to 8 mg/kg TCS did not produce the cell apoptosis in the labyrinth zone (data not shown).

TCS-induced reductions in fetal body weight occur after GD11. Fetal body weight correlates positively with placental development and nutrient transfer capacity during mid-late gestation. Placental hypoplasia, particularly a reduced labyrinth zone volume, was observed in 8-TCS mice. During rat and mouse placental development, cell proliferative activity peaks in the basal zone and metrial gland on GD11 and GD12, while in the labyrinth zone on GD13^24^. The cell proliferative capability of the labyrinth zone is greater and lasts longer than that of its counterparts. By counting BrdU^+^ cells and PCNA immunohistochemistry on GD13, we found that cell proliferative capability in the labyrinth zone was significantly reduced in TCS-8 mice. The idea is supported by the low expression of the proliferation markers PCNA and Cyclin D3 in placentas of TCS-8 mice compared to control mice placentas. The expression of PCNA, as a marker for the cell cycle, peaks in late G1 and S phases of the cell cycle, which is necessary for deoxyribonucleic acid (DNA) synthesis in mammalian cells^33^. The Cyclin D3 takes role in transition from G1 to S phase of the cell cycle. Thus, it is indicated that the decreased PCNA and Cyclin D3 may be one of the possible reasons of placental hypoplasia in TCS-8 mice. Activation of thyroid hormones receptors, which can function as nuclear transcription factors, regulates gene transcription. The hypothyroidism reduces approximately 25% PCNA in spermatogonia^34^. Hypothyroxinemia may contribute to the pathophysiology of placental hypoplasia^35^. The thyroid hormone rapidly activates the Akt-mTOR signaling pathways^36^. The activation of mTOR induces trophoblast cell proliferation^37^. The down-regulation of placental Akt-mTOR-P70S6K signaling in 8-TCS mice was corrected by the administration of T4. Furthermore, T4 replacement recovered placental cell proliferative capability and the expression of PCNA and Cyclin D3 in 8-TCS mice, which was blocked by mTOR inhibition. Although T4 and T3 are known to induce ERK-dependent cell proliferation by reducing the expression of genes that inhibit the cell cycle^38^, the placental ERK activity was not altered in 8-TCS mice. Thus, these results indicate that TCS-induced hypothyroxinemia through reducing Akt and mTOR activities may impair placental growth and development. It is widely accepted that optimal maternal thyroid hormone concentrations can play a critical role in maintaining a balanced inflammatory response in early pregnancy to prevent fetal immune rejection and promote normal placental development through the regulation of the secretion of critical cytokines^39^. The maternal thyroid dysfunction during early pregnancy is associated with complications of miscarriage and pre-eclampsia. A recent study has reported that in the first trimester T3 reduces the secretion of IL-1β and IL-10 and increases the secretion of tumour necrosis factor-α (TNF-α) and IL-6, suggesting a role of T3 in the regulation of the immune balance at the uteroplacental interface^40^. In contrast, in the second trimester T3 increases only IL-10 secretion, but does not affect the secretion of other cytokines. We observed that the levels of placental inflammatory factors IL-6, IL-1β and TNF-α on GD17 had no significant difference between control mice and TCS-mice (Supplemental Fig. 2a–c).

The activities of placental amino acid and glucose transporters were reduced in 8-TCS mice, although the accumulation of [^14^C]-MeAIB and [^14^C]-glucose accumulation per gram of fetus was not altered in these mice. The above mentioned decreases in the activities of amino acid and glucose transporters were recovered by the T4 replacement, indicating that TCS-induced hypothyroxinemia suppresses the placental nutrient transporter function. Placental mTOR inhibition decreases cell surface amino acid transporter abundance, while mTOR activation increases cell surface amino acid transporter abundance in trophoblasts^20^. The inhibition of mTOR by rapamycin significantly reduces the activities of system A, system L and TAUT transporters^41^. The inhibition of PI3K or mTOR effectively reduces the GLUT1 membrane localization^21^. The mTORC1 or mTORC2 silencing markedly decreases the plasma membrane expression of System A and System L transporters^22^. Thus, it is conceivable that TCS-induced hypothyroxinemia through the down-regulated Akt-mTOR-P70S6K signaling may reduce the activities of amino acid and glucose transporters. In addition, the mTOR can regulate the posttranslational modifications of placental amino acid transporters and transporter translocation to plasma membrane^41^. Placental SNAT1 and SNAT4 expression was decreased in 8-TCS mice, which was recovered by the T4 replacement in an mTOR-dependent manner. Maternal hypothyroxinemia reportedly reduces GLUT1 expression and increases GLUT3 protein expression^42^. Interestingly, the T4 replacement in 8-TCS mice corrected the decrease in placental GLUT1 expression. This effect was not sensitive to rapamycin. Thus, additional works are required to explore the mTOR-independent mechanisms underlying hypothyroidism-reduced GLUT1 expression.

Several lines of evidence suggest the placental morphological and functional adaptation^15^,^27^. When the placental mass is reduced, the placental amino acids transport is enhanced, at least in part, through increased expression of the transporter genes Slc2a3 and Slc38a4. This adaptability in placental phenotype provides a functional reserve capacity to better match the placental nutrient supply with the fetal nutrient demands. We in the present study observed that the placental size and the placental nutrient transfer were reduced in 8-TCS mice compared to controls. The T4 replacement on GD10-13 in 8-TCS mice could rescue the cell proliferative activity to protect the placental development, but it failed to perfectly recover the activities of amino acid and glucose transporters on GD17 (data not shown). By contrast, the T4 replacement on GD15-17 significantly improved the activities of amino acid transporter and glucose transporter in 8-TCS mice. The placenta can respond to fetal demand signals through regulating expression of placental transport systems^43^. mTOR functions as an important placental growth signaling sensor^37^. The T4-recovered amino acid and glucose transporter function in 8-TCS mice were sensitive to the inhibition of mTOR. Therefore, the findings indicate that the deficits in the placental nutrient transfer are caused by the TCS-induced suppression of transporters expression and the disruption of nutrient sensors to fetal demand signals.

TCS is widely used in personal care products. The present study provides *in vivo* evidence that exposing pregnant mice to 8 mg/kg TCS causes reductions in thyroid hormone levels resulting in Akt-mTOR-P70S6K signaling down-regulation, which affects placental development and nutrient transport and eventually leads to decreases in fetal body weight.

## Materials and Methods

### Animals and drug administration

All animal experimental procedures followed the guidelines of the Laboratory Animal Research of Nanjing Medical University and were approved by the Institutional Animal Care and Use Committee of Nanjing Medical University. Three-month-old female and male mice (Oriental Bioservice Inc., Nanjing), weighing approximately 30–35 g, were used in this study. All of the mice were housed under a 12/12 hour light/dark cycle (lights on at 0600 hour) with free access to food and tap water. Vaginal plug detection was chosen as the indicator of gestational day (GD) 1. TCS (Sigma-Aldrich Inc., St. Louis, Mo.) was dissolved in dimethylsulfoxide (DMSO), and then diluted with corn oil. The dams were given oral administration of TCS at doses of 1, 4 and 8 mg/kg per day. These doses were chosen based on a recent report that mice exposed to 10 mg/kg TCS exhibited urinary TCS levels equivalent to those of spontaneous abortion patients^5^. Levothyroxine (T4) (Sigma-Aldrich Inc., St. Louis, MO, USA) was dissolved in 0.9% saline solution and injected (20 μg/kg/day) subcutaneously (s.c.)^44^. The mTOR inhibitor rapamycin (Sigma-Aldrich Inc., St Louis, MO, USA) and the PI3K inhibitor LY294002 (Sigma-Aldrich Inc., St. Louis, MO, USA) were dissolved in 0.9% saline solution. Rapamycin was injected intraperitoneally (i.p.) at a dose of 3.5 mg/kg/day^45^. LY294002 (40 μM) was administered by intrauterine injections^46^. The mice were deeply anesthetized, and an incision was made in the lower abdomen. Each horn was divided into three equal parts: the cervical one third, the central one third, and the ovarian one third^47^. The solution of LY294002 (10 μl) was injected into the luminal space of each uterine horn by the following sequence: central portion, cervical portion and portion of ovarian side. The incision was then closed, and the mice were returned to their cages. Control mice were treated with identical volumes of vehicle.

### Histological placental examination

The placentas were fixed in 4% paraformaldehyde and then dehydrated using a graded series of alcohol, cleared in xylene and embedded in paraffin wax. Sections (5 μm) were deparaffinized and stained with hematoxylin and eosin (HE). Detailed structural analyses of the labyrinthine zone were performed using a conventional light microscope (Olympus DP70; Tokyo, Japan). The Computer Assisted Stereological Toolbox (CAST v2.0) was employed to superimpose grids and generate random fields of view within systematic random paraffin sections. Placental volumes densities were measured using a point grid and the following equation: V(obj) = t × Σa = t × a(p) × ΣP, which was used to convert volume densities into absolute values, where V(obj) is the estimated placental volume, t is the total thickness of the placenta, a(p) is the area associated with each point and ΣP is the mean number of points per section. The volumes of the labyrinthine zone were determined by point counting and then converting the volume densities into absolute values^48^. The volume fractions of the labyrinthine zone represent the percentage of the entire placenta occupied by the labyrinthine zone.

### Cell proliferation examination

BrdU (Sigma-Aldrich) was dissolved freshly in 0.9% saline to form a 10 mg/ml solution just before injection. To assay placental cell proliferation, we injected pregnant female mice with BrdU (100 mg/kg body weight), sacrificed the mice at 3 h after injection and fixed their placentas in 4% paraformaldehyde overnight. Briefly, the paraffin embedded sections (5 μm) were mounted on positively charged slides and incubated overnight with anti-BrdU antibody (1:1000, Millipore, Billerica, MA, USA) or anti-PCNA antibody (1:500, Millipore, Billerica, MA, USA) at 4 °C. The sections were incubated with biotin-labeled goat anti-mouse IgG antibodies (1:500, Bioworld Technology, Inc., St. Louis Park, MN, USA) for 2 h. Immunoreactivity was visualized using an avidin-biotin horseradish peroxidase complex (Vector Laboratories, Inc., Burlingame, CA, USA). The numbers of BrdU-positive (BrdU^+^) cells in the labyrinthine zone of every 5^th^ section (25 μm apart) were counted using a conventional light microscope (DP70, Olympus Optical, Tokyo, Japan).

### Placental amino acid and glucose transporter activity measurements

Unidirectional materno-fetal transfer of non-metabolizable radioactive tracers was measured as described by Coan *et al*.^27^. Briefly, pregnant mice were anesthetized with an intraperitoneal injection (0.4 ml) of fentanyl/fluanisone and midazolam solutions in water (1:1:2 water, Janseen Animal Health). After the maternal jugular vein was exposed, a 100 μl bolus of PBS containing 3.5 μCi (1 Ci = 37GBq) of [^14^C]methyl amino-isobutyrate (MeAIB) (specific activity 50.5 mCi/mmol) or 3.5 μCi of [^14^C]-methyl-D-glucose (specific activity 56.4 mCi/mmol) was injected into the jugular vein *via* a short length of tubing attached to a 27 gauge needle and connected to a 1 ml syringe. At times up to 4 min after tracer injection, the mice were killed and their conceptuses were dissected via hysterectomy. The fetuses were lysed overnight in 2 ml of Biosol (National Diagnostics) at 55 °C. Fetal sample fractions were then added to the appropriate tubes for β counting (Packard Tri-Carb 1900). The radioactive counts of each fetus were used to calculate the amount of radioisotope transferred per gram of placenta or fetus.

### Serum hormone measurements

Orbital blood samples were obtained from GD19 dams under anesthetized conditions with pentobarbital (3 mg/100 ml, i.p.). The serum was separated by centrifugation at 4 °C and stored at −80 °C until assay. Total T4 and T3 levels, as well as TSH, E2 and P4 levels were measured using commercial enzyme-linked immunosorbent assay (ELISA) kits (Uscn Life Science Inc., Houston, USA), according to the munafacturer’s instructions. The sensitivities were 1.4 ng/ml for T4, 47.1 pg/ml for T3, 19.3 pg/ml for TSH, 2.0 pg/ml for E2, and 0.2 ng/ml for P4. The intra- and inter-assay coefficients of variation were 4.3% and 7.5% for T4, 4.5% and 7.2% for T3, 3.2% and 9.5% for TSH, 6.0% and 5.8% for E2, and 5.8% and 8.4% for P4.

### Placental inflammatory factors measurements

Placentas were removed by cesarean section and then quickly frozen and kept at −80 °C until extraction. Proteins were extracted as previously described^49^. Commercial ELISA (Uscn Life Science Inc., Houston, USA) kits were used to determine levels of IL-1β and IL-6 according to the manufacturer’s protocol.

### Western blot analysis

Western blot analysis was performed as described previously^5^. Briefly, the placenta was homogenized to obtain protein samples. Then, the proteins (50 μg) were resolved by SDS-PAGE, transferred onto PVDF membranes, and probed with anti-phosphorylation antibodies to Akt (1:1000, Cell Signaling Technology, Inc., USA), mTOR (1:1000, Cell Signaling), p70S6K (1:1000, Bioworld Technology), ERK1/2 (1:1000, Cell Signaling) and antibody of TNF-α (1:500, Bioworld Technology) at 4 °C overnight. The membranes were then incubated with an HRP-labeled secondary antibody and developed using an ECL detection kit (Millipore, MA, USA). Following visualization, the blots were stripped by incubation in stripping buffer (Restore, Pierce) for 5 min, and then incubated with antibodies to Akt (1:1000, Cell Signaling), mTOR (1:1000, Cell Signaling), p70S6K (1:1000, Bioworld Technology), ERK1/2 (1:1000, Cell Signaling) and β-actin (1:1000, Cell Signaling). Western blot bands were scanned and analyzed with the National Institutes of Health Image image analysis software package.

### Reverse transcription quantitative polymerase chain reaction (RT-qPCR)

Total RNA was extracted from the placentas using Trizol (Invitrogen, Carlsbad, CA, USA) according to the manufacturer’s instructions. RNA (1 μg) was used for reverse transcription using high-capacity cDNA of the reverse transcription kit RT (TaKaRa Biotechnology CO., Ltd) according to the manufacturer’s instructions. The *PCNA, Cyclin D3, Slc38a1/SNAT1, Slc38a2/SNAT2, Slc38a4/SNAT4, TAUT, Slc2a1/GLUT1, Slc2a3/GLUT3* and *GAPDH* mRNA primer sequences were designed according to earlier publications^15^,^50^,^51^. RT-qPCR was performed using a Light Cycler Fast Start DNA Master SYBR Green I Kit and an ABI Prism 7300 Sequence Detection System (Applied Biosystems, Foster City, California, USA), and relative gene expression was determined using the 2-ΔΔct method with normalization to GAPDH expression. The results were averaged from four sets of independent experiments.

### Statistical analysis

Group data are expressed as the mean ± standard error (SE). All of the statistical analyses were performed using SPSS 16.0 software (SPSS Inc., Chicago, IL, USA). Differences among means were analyzed using one/two-factor analysis of variance (ANOVA) followed by Bonferroni *post hoc* analysis. Differences at *P* < 0.05 were considered statistically significant.

## Additional Information

**How to cite this article:** Cao, X. *et al*. Exposure of pregnant mice to triclosan impairs placental development and nutrient transport. *Sci. Rep.* **7**, 44803; doi: 10.1038/srep44803 (2017).

**Publisher's note:** Springer Nature remains neutral with regard to jurisdictional claims in published maps and institutional affiliations.

## Supplementary Material

Supplementary Information

## Figures and Tables

**Table 1:** Activity of placental amino acid and glucose transporters on GD14 and GD17.

	Control	TCS (mg/kg)			control	TCS (mg/kg)		
		1	4	8		1	4	8
	GD14				GD17			
MeAIB d.p.m./g*10^5^ (placenta)	0.292 ± 0.015	0.281 ± 0.022	0.277 ± 0.016	0.248 ± 0.014*	1.72 ± 0.09	1.65 ± 0.10	1.61 ± 0.15	1.33 ± 0.12**
MeAIB d.p.m./g*10^5^ (fetus)	0.138 ± 0.009	0.145 ± 0.009	0.148 ± 0.008	0.147 ± 0.009	0.96 ± 0.08	0.93 ± 0.12	0.94 ± 0.13	0.94 ± 0.09
glucose d.p.m./g*10^6^ (placenta)	0.446 ± 0.017	0.430 ± 0.020	0.420 ± 0.022	0.389 ± 0.019*	1.72 ± 0.08	1.64 ± 0.13	1.62 ± 0.14	1.44 ± 0.10*
glucose d.p.m./g*10^6^ (fetus)	0.194 ± 0.008	0.188 ± 0.010	0.193 ± 0.011	0.181 ± 0.012	0.97 ± 0.08	0.96 ± 0.09	0.91 ± 0.05	0.94 ± 0.07

*P < 0.05 and **P < 0.01 *vs*. controls (one-way ANOVA).

**Table 2:** Levels of placental amino acid and glucose transporters mRNA on GD14 and GD17.

mRNA	control	TCS (mg/kg)			control	TCS (mg/kg)			TCS/T4	TCS/T4/Re
		1	4	8		1	4	8	8	8
	GD14				GD17					
*SNAT1*	1.00 ± 0.07	0.94 ± 0.12	0.92 ± 0.06	0.80 ± 0.06*	1.00 ± 0.03	0.96 ± 0.07	0.92 ± 0.06	0.67 ± 0.11**	0.93 ± 0.06^#^	0.66 ± 0.11^+^
*SNAT2*	1.00 ± 0.05	0.98 ± 0.07	0.94 ± 0.08	0.90 ± 0.08	1.00 ± 0.05	0.93 ± 0.06	0.89 ± 0.06	0.86 ± 0.07	0.88 ± 0.07	0.83 ± 0.09
*SNAT4*	1.00 ± 0.07	0.92 ± 0.05	0.89 ± 0.06	0.83 ± 0.05*	1.00 ± 0.04	0.93 ± 0.06	0.88 ± 0.06	0.69 ± 0.06**	0.85 ± 0.04^#^	0.71 ± 0.04^+^
*GLUT1*	1.00 ± 0.06	0.94 ± 0.07	0.91 ± 0.08	0.87 ± ± 0.05*	1.00 ± 0.04	0.97 ± 0.02	0.94 ± 0.04	0.89 ± 0.05*	1.02 ± 0.03^#^	0.95 ± 0.13
*GLUT3*	1.00 ± 0.07	0.94 ± 0.08	0.93 ± 0.07	0.94 ± 0.08	1.00 ± 0.04	0.98 ± 0.09	0.95 ± 0.07	0.93 ± 0.08	0.94 ± 0.08	0.93 ± 0.09

*P < 0.05 and **P < 0.01 *vs*. controls; ^#^*P* < 0.05 *vs*. 8-TCS mice; ^+^*P* < 0.05 *vs*. 8-TCS+T4 mice (two-way ANOVA).

**Table 3:** Levels of thyroid hormones and reproductive hormones on GD17.

Hormones	Control	TCS (mg/kg)		
		1	4	8
T4 (ng/ml)	34.46 ± 1.62	33.4 ± 2.20	31.59 ± 2.13	28.40 ± 1.48**
T3 (ng/ml)	1.48 ± 0.07	1.39 ± 0.06	1.33 ± 0.10	1.09 ± 0.12**
TSH (uIU/ml)	1.24 ± 0.11	1.34 ± 0.08	1.46 ± 0.08	1.59 ± 0.13*
E2 (pg/ml)	33.06 ± 1.63	32.84 ± 1.60	32.44 ± 2.00	32.04 ± 2.02
P4 (pg/ml)	25.21 ± 0.79	24.54 ± 0.95	23.03 ± 1.08	22.7 ± 1.02

*P < 0.05 and **P < 0.01 *vs*. controls (one-way ANOVA).

## References

[b1] Dhillon G. S. . Triclosan: current status, occurrence, environmental risks and bioaccumulation potential. International journal of environmental research and public health 12, 5657–5684 (2015). 26006133 10.3390/ijerph120505657 PMC4454990

[b2] Allmyr M., Adolfsson-Erici M., McLachlan M. S. & Sandborgh-Englund G. Triclosan in plasma and milk from Swedish nursing mothers and their exposure via personal care products. The Science of the total environment 372, 87–93 (2006). 17007908 10.1016/j.scitotenv.2006.08.007

[b3] Toms L. M. . Triclosan in individual human milk samples from Australia. Chemosphere 85, 1682–1686 (2011). 22000243 10.1016/j.chemosphere.2011.08.009

[b4] Pycke B. F. . Human fetal exposure to triclosan and triclocarban in an urban population from Brooklyn, New York. Environ Sci Technol 48, 8831–8838 (2014). 24971846 10.1021/es501100w PMC4123932

[b5] Wang X. . Triclosan causes spontaneous abortion accompanied by decline of estrogen sulfotransferase activity in humans and mice. Scientific reports 5, 18252 (2015). 26666354 10.1038/srep18252 PMC4678904

[b6] James M. O., Li W., Summerlot D. P., Rowland-Faux L. & Wood C. E. Triclosan is a potent inhibitor of estradiol and estrone sulfonation in sheep placenta. Environment international 36, 942–949 (2010). 19299018 10.1016/j.envint.2009.02.004 PMC4789100

[b7] Crofton K. M., Paul K. B., Devito M. J. & Hedge J. M. Short-term *in vivo* exposure to the water contaminant triclosan: Evidence for disruption of thyroxine. Environmental toxicology and pharmacology 24, 194–197 (2007). 21783810 10.1016/j.etap.2007.04.008

[b8] Witorsch R. J. Critical analysis of endocrine disruptive activity of triclosan and its relevance to human exposure through the use of personal care products. Critical reviews in toxicology 44, 535–555 (2014). 24897554 10.3109/10408444.2014.910754

[b9] Rodriguez P. E. & Sanchez M. S. Maternal exposure to triclosan impairs thyroid homeostasis and female pubertal development in Wistar rat offspring. *Journal of toxicology and environmental health*. Part A 73, 1678–1688 (2010). 10.1080/15287394.2010.516241 21058171

[b10] Saki F. . Thyroid function in pregnancy and its influences on maternal and fetal outcomes. International journal of endocrinology and metabolism 12, e19378 (2014). 25745488 10.5812/ijem.19378 PMC4338651

[b11] Chen L. M. . Effects of subclinical hypothyroidism on maternal and perinatal outcomes during pregnancy: a single-center cohort study of a Chinese population. PloS one 9, e109364 (2014). 25353960 10.1371/journal.pone.0109364 PMC4212915

[b12] Fowden A. L., Ward J. W., Wooding F. P., Forhead A. J. & Constancia M. Programming placental nutrient transport capacity. The Journal of physiology 572, 5–15 (2006). 16439433 10.1113/jphysiol.2005.104141 PMC1779642

[b13] Higgins L., Greenwood S. L., Wareing M., Sibley C. P. & Mills T. A. Obesity and the placenta: A consideration of nutrient exchange mechanisms in relation to aberrant fetal growth. Placenta 32, 1–7 (2011). 21030077 10.1016/j.placenta.2010.09.019

[b14] Brett K. E., Ferraro Z. M., Yockell-Lelievre J., Gruslin A. & Adamo K. B. Maternal-fetal nutrient transport in pregnancy pathologies: the role of the placenta. International journal of molecular sciences 15, 16153–16185 (2014). 25222554 10.3390/ijms150916153 PMC4200776

[b15] Constancia M. . Adaptation of nutrient supply to fetal demand in the mouse involves interaction between the Igf2 gene and placental transporter systems. Proceedings of the National Academy of Sciences of the United States of America 102, 19219–19224 (2005). 16365304 10.1073/pnas.0504468103 PMC1316882

[b16] Illsley N. P. Glucose transporters in the human placenta. Placenta 21, 14–22 (2000). 10692246 10.1053/plac.1999.0448

[b17] Santalucia T., Palacin M. & Zorzano A. T3 strongly regulates GLUT1 and GLUT3 mRNA in cerebral cortex of hypothyroid rat neonates. Molecular and cellular endocrinology 251, 9–16 (2006). 16581179 10.1016/j.mce.2006.02.016

[b18] Scarlett A. . Thyroid hormone stimulation of extracellular signal-regulated kinase and cell proliferation in human osteoblast-like cells is initiated at integrin alphaVbeta3. The Journal of endocrinology 196, 509–517 (2008). 18310446 10.1677/JOE-07-0344

[b19] Barbakadze T., Natsvlishvili N. & Mikeladze D. Thyroid hormones differentially regulate phosphorylation of ERK and Akt via integrin alphavbeta3 receptor in undifferentiated and differentiated PC-12 cells. Cell biochemistry and function 32 (2014). 10.1002/cbf.3013 24214887

[b20] Roos S., Powell T. L. & Jansson T. Placental mTOR links maternal nutrient availability to fetal growth. Biochemical Society transactions 37, 295–298 (2009). 19143650 10.1042/BST0370295

[b21] Makinoshima H. . Signaling through the Phosphatidylinositol 3-Kinase (PI3K)/Mammalian Target of Rapamycin (mTOR) Axis Is Responsible for Aerobic Glycolysis mediated by Glucose Transporter in Epidermal Growth Factor Receptor (EGFR)-mutated Lung Adenocarcinoma. The Journal of biological chemistry 290, 17495–17504 (2015). 26023239 10.1074/jbc.M115.660498 PMC4498084

[b22] Rosario F. J., Kanai Y., Powell T. L. & Jansson T. Mammalian target of rapamycin signalling modulates amino acid uptake by regulating transporter cell surface abundance in primary human trophoblast cells. The Journal of physiology 591, 609–625 (2013). 23165769 10.1113/jphysiol.2012.238014 PMC3577540

[b23] Crawford B. R. & Decatanzaro D. Disruption of blastocyst implantation by triclosan in mice: impacts of repeated and acute doses and combination with bisphenol-A. Reproductive toxicology 34, 607–613 (2012). 23059059 10.1016/j.reprotox.2012.09.008

[b24] Coan P. M., Conroy N., Burton G. J. & Ferguson-Smith A. C. Origin and characteristics of glycogen cells in the developing murine placenta. Developmental dynamics: an official publication of the American Association of Anatomists 235, 3280–3294 (2006). 17039549 10.1002/dvdy.20981

[b25] Acar N. . Is there a relationship between PCNA expression and diabetic placental development during pregnancy? Acta histochemica 110, 408–417 (2008). 18377963 10.1016/j.acthis.2007.11.011

[b26] Constancia M. . Placental-specific IGF-II is a major modulator of placental and fetal growth. Nature 417, 945–948 (2002). 12087403 10.1038/nature00819

[b27] Coan P. M. . Adaptations in placental nutrient transfer capacity to meet fetal growth demands depend on placental size in mice. The Journal of physiology 586, 4567–4576 (2008). 18653658 10.1113/jphysiol.2008.156133 PMC2614013

[b28] Feng Y. . Endocrine Disrupting Effects of Triclosan on the Placenta in Pregnant Rats. PloS one 11, e0154758 (2016). 27149376 10.1371/journal.pone.0154758 PMC4858197

[b29] Fang J. L. . Dose–response assessment of the dermal toxicity of triclosan in B6C3F1 mice. Toxicology Research 4, 867–877 (2015).

[b30] Zorrilla L. M. . The effects of triclosan on puberty and thyroid hormones in male Wistar rats. Toxicological sciences: an official journal of the Society of Toxicology 107, 56–64 (2009). 18940961 10.1093/toxsci/kfn225

[b31] Wu Y., Beland F. A. & Fang J. L. Effect of triclosan, triclocarban, 2,2′,4,4′-tetrabromodiphenyl ether, and bisphenol A on the iodide uptake, thyroid peroxidase activity, and expression of genes involved in thyroid hormone synthesis. Toxicology in vitro: an international journal published in association with BIBRA 32, 310–31 (2016). 26827900 10.1016/j.tiv.2016.01.014

[b32] Paul K. B., Hedge J. M., DeVito M. J. & Crofton K. M. Short-term exposure to triclosan decreases thyroxine *in vivo* via upregulation of hepatic catabolism in Young Long-Evans rats. Toxicological sciences: an official journal of the Society of Toxicology 113, 367–379 (2010). 19910387 10.1093/toxsci/kfp271 PMC2902919

[b33] Braun T. . Maternal betamethasone administration reduces binucleate cell number and placental lactogen in sheep. The Journal of endocrinology 194, 337–347 (2007). 17641283 10.1677/JOE-07-0123

[b34] Tousson E., Ali E. M., Ibrahim W. & Mansour M. A. Proliferating cell nuclear antigen as a molecular biomarker for spermatogenesis in PTU-induced hypothyroidism of rats. Reproductive sciences 18, 679–686 (2011). 21273639 10.1177/1933719110395401

[b35] Vasilopoulou E. . Differential triiodothyronine responsiveness and transport by human cytotrophoblasts from normal and growth-restricted pregnancies. The Journal of clinical endocrinology and metabolism 95, 4762–4770 (2010). 20660035 10.1210/jc.2010-0354 PMC3050105

[b36] Kenessey A. & Ojamaa K. Thyroid hormone stimulates protein synthesis in the cardiomyocyte by activating the Akt-mTOR and p70S6K pathways. The Journal of biological chemistry 281, 20666–20672 (2006). 16717100 10.1074/jbc.M512671200

[b37] Wen H. Y., Abbasi S., Kellems R. E. & Xia Y. mTOR: a placental growth signaling sensor. Placenta 26 Suppl A, S63–69 (2005). 15837070 10.1016/j.placenta.2005.02.004

[b38] Shinderman-Maman E. . The thyroid hormone-alphavbeta3 integrin axis in ovarian cancer: regulation of gene transcription and MAPK-dependent proliferation. Oncogene 35, 1977–1987 (2016). 26165836 10.1038/onc.2015.262

[b39] Challis J. R. . Inflammation and pregnancy. Reproductive sciences 16, 206–215 (2009). 19208789 10.1177/1933719108329095

[b40] Vasilopoulou E. . Triiodothyronine regulates angiogenic growth factor and cytokine secretion by isolated human decidual cells in a cell-type specific and gestational age-dependent manner. Human reproduction 29, 1161–1172 (2014). 24626803 10.1093/humrep/deu046 PMC4017942

[b41] Roos S., Kanai Y., Prasad P. D., Powell T. L. & Jansson T. Regulation of placental amino acid transporter activity by mammalian target of rapamycin. American journal of physiology. Cell physiology 296, C142–150 (2009). 18987252 10.1152/ajpcell.00330.2008

[b42] Pickard M. R. . Maternal hypothyroxinemia influences glucose transporter expression in fetal brain and placenta. The Journal of endocrinology 163, 385–394 (1999). 10588811 10.1677/joe.0.1630385

[b43] Jansson T. & Powell T. L. IFPA (2005) Award in Placentology Lecture. Human placental transport in altered fetal growth: does the placenta function as a nutrient sensor? – a review. Placenta 27 Suppl A, S91–97 (2006). 16442615 10.1016/j.placenta.2005.11.010

[b44] Wang S. . Early levothyroxine treatment on maternal subclinical hypothyroidism improves spatial learning of offspring in rats. Journal of neuroendocrinology 24, 841–848 (2012). 22192600 10.1111/j.1365-2826.2011.02275.x

[b45] Festuccia W. T. . PPARgamma activation attenuates glucose intolerance induced by mTOR inhibition with rapamycin in rats. American journal of physiology. Endocrinology and metabolism 306, E1046–1054 (2014). 24619883 10.1152/ajpendo.00683.2013

[b46] Liu L., Wang Y. & Yu Q. The PI3K/Akt signaling pathway exerts effects on the implantation of mouse embryos by regulating the expression of RhoA. International journal of molecular medicine 33, 1089–1096 (2014). 24638941 10.3892/ijmm.2014.1701 PMC4020477

[b47] Zhu L. J., Bagchi M. K. & Bagchi I. C. Attenuation of calcitonin gene expression in pregnant rat uterus leads to a block in embryonic implantation. Endocrinology 139, 330–339 (1998). 9421431 10.1210/endo.139.1.5707

[b48] Coan P. M., Ferguson-Smith A. C. & Burton G. J. Developmental dynamics of the definitive mouse placenta assessed by stereology. Biology of reproduction 70, 1806–1813 (2004). 14973263 10.1095/biolreprod.103.024166

[b49] Palei A. C., Spradley F. T. & Granger J. P. Euglycemic hyperinsulinemia increases blood pressure in pregnant rats independent of placental antiangiogenic and inflammatory factors. American journal of hypertension 26, 1445–1451 (2013). 23955606 10.1093/ajh/hpt137 PMC3816628

[b50] Gupta P., Bhatia N., Bansal M. P. & Koul A. Lycopene modulates cellular proliferation, glycolysis and hepatic ultrastructure during hepatocellular carcinoma. World journal of hepatology 8, 1222–1233 (2016). 27803767 10.4254/wjh.v8.i29.1222 PMC5067442

[b51] Junyent F. . Content and traffic of taurine in hippocampal reactive astrocytes. Hippocampus 21, 185–197 (2011). 20082296 10.1002/hipo.20739

