# Supplementary material for: Exposure of pregnant mice to triclosan impairs placental development and nutrient transport
Source: Sci Rep. 2017 Mar 21;7:44803. doi: 10.1038/srep44803 (PMC5359620; doi:10.1038/srep44803)
Supplement: Supplementary Information [file srep44803-s1.doc]

**Supplementary Information**

**Exposure of pregnant mice to triclosan impairs placental development and nutrient transport**

Short title: TCS-impaired placental development and function

Xinyuan Cao^a^, Xu Hua^b^, *Xiaoli Wang^b,c^, *Ling Chen^a,b^

^a^State Key Lab of Reproductive Medicine, ^b^Department of Physiology, ^c^Department of Pharmacology, Nanjing Medical University, Nanjing 211166, China,

*Corresponding author: Ling Chen, Ph.D. & M.D or Xiaoli Wang, Ph.D.

Address: Department of Physiology, Nanjing Medical University, Longmian Road 101, Nanjing, China.

Tel: +86-25-86869441, Fax: +86-25-86260332

E-mail: [lingchen@njmu.edu.cn](mailto:lingchen@njmu.edu.cn) and wang130560@njmu.edu.cn

**Supplemental Figure 1**

**
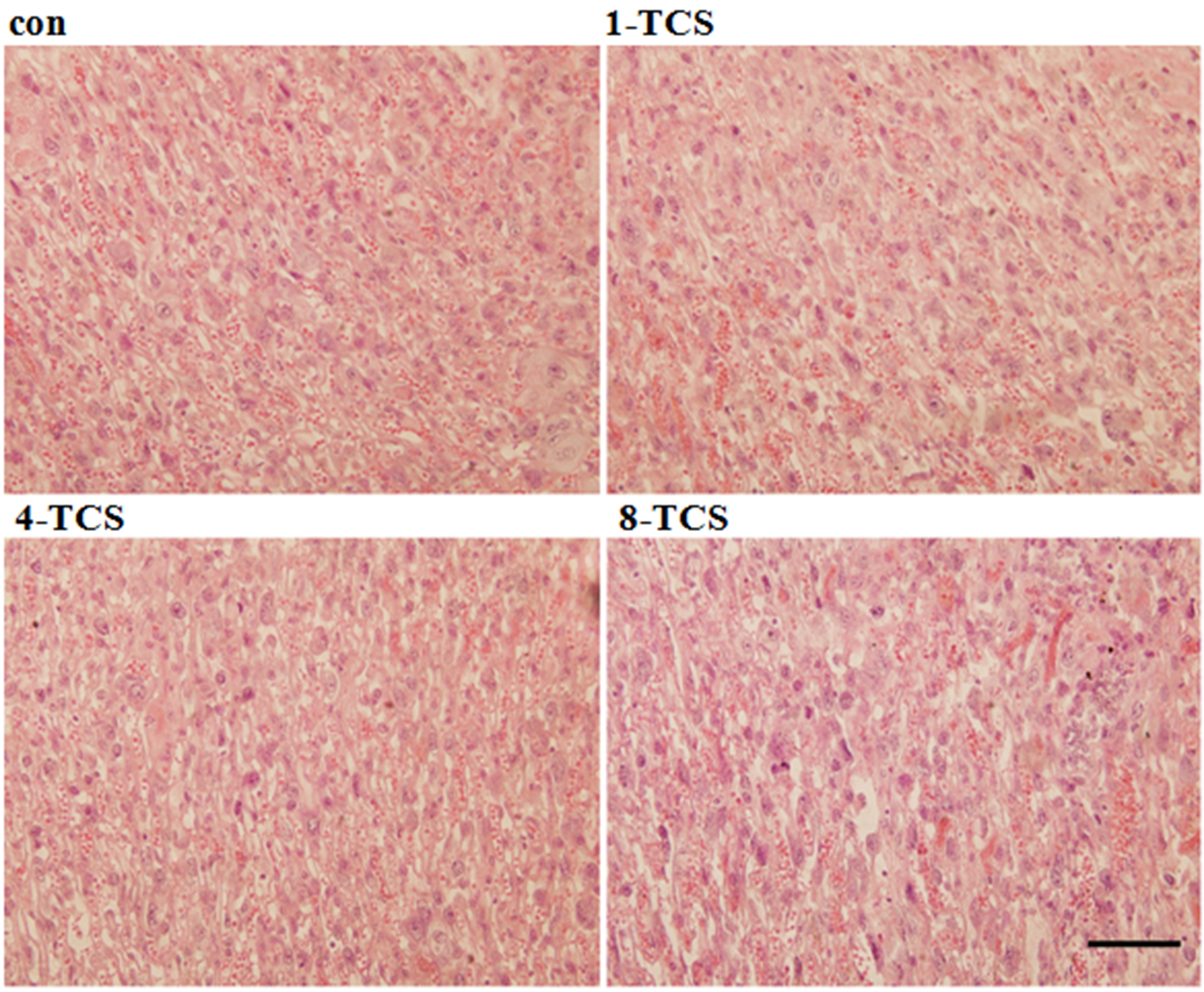
**

**Supplemental Figure 1** Representative picture of placental labyrinthine zone stained with hematoxylin and eosin on GD19. Scale bars = 50 μ m.

**Supplemental Figure 2a-c**

**
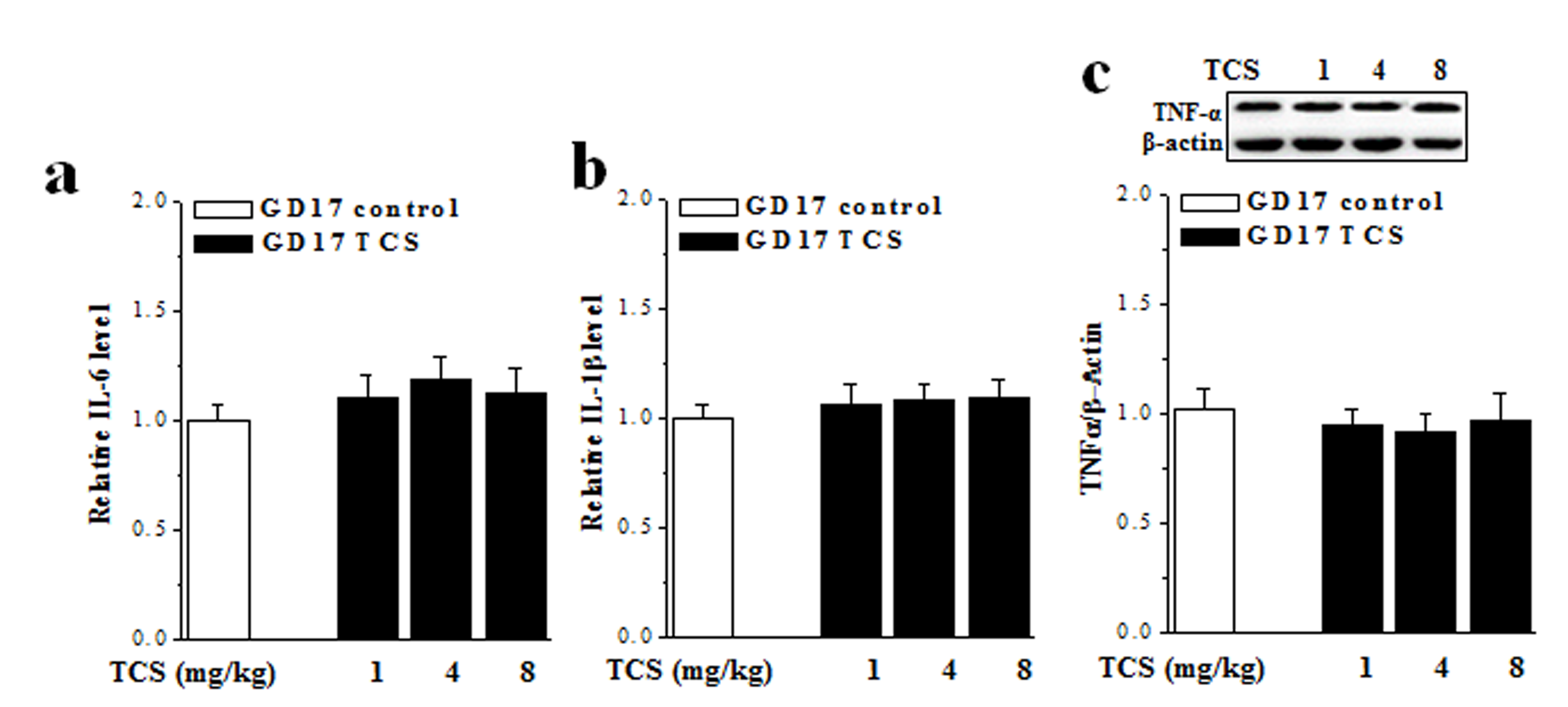
**

**Supplemental Figure 2** Effects of TCS on the levels of placental inflammatory factors IL-6 (**a**), IL-1β (**b**) and TNF-α (**c**) on GD17.

**Supplemental Figure 3a-d**

**
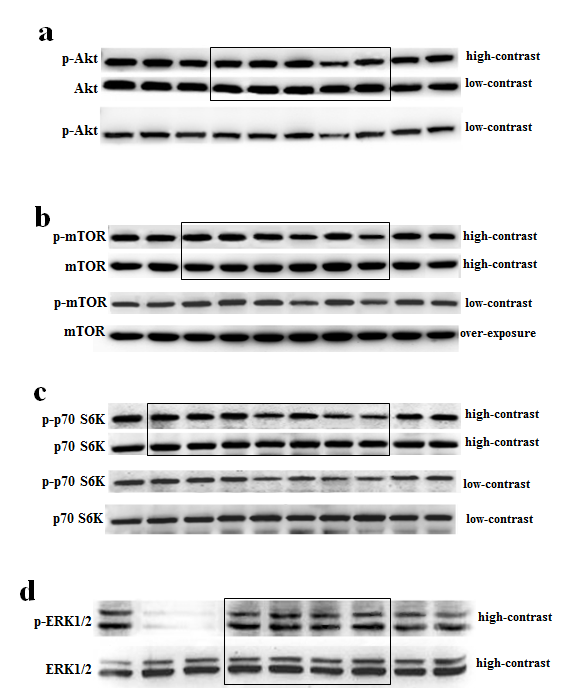
**

**Supplemental Figure 3** Full-length immunoblots of Akt (**a**), mTOR (**b**), p70S6K (**c**) and ERK1/2 (**d**) phosphorylation (upper) and protein (bottom) on GD17. The regions of line rectangles in these full-length blots/gels indicate that the representative images used in ***Figure 3a-d*** of the main text.
